# An Update on CRF Mechanisms Underlying Alcohol Use Disorders and Dependence

**DOI:** 10.3389/fendo.2016.00134

**Published:** 2016-10-21

**Authors:** Isabel Marian Hartmann Quadros, Giovana Camila Macedo, Liz Paola Domingues, Cristiane Aparecida Favoretto

**Affiliations:** ^1^Department of Psychobiology, Escola Paulista de Medicina, Universidade Federal de São Paulo, São Paulo, São Paulo, Brazil

**Keywords:** alcohol, addiction, alcohol self-administration, neuropeptides, animal models, mesocorticolimbic system, sensitization, alcohol-related aggression

## Abstract

Alcohol is the most commonly used and abused substance worldwide. The emergence of alcohol use disorders, and alcohol dependence in particular, is accompanied by functional changes in brain reward and stress systems, which contribute to escalated alcohol drinking and seeking. Corticotropin-releasing factor (CRF) systems have been critically implied in the transition toward problematic alcohol drinking and alcohol dependence. This review will discuss how dysregulation of CRF function contributes to the vulnerability for escalated alcohol drinking and other consequences of alcohol consumption, based on preclinical evidence. CRF signaling, mostly *via* CRF1 receptors, seems to be particularly important in conditions of excessive alcohol taking and seeking, including during early and protracted withdrawal, relapse, as well as during withdrawal-induced anxiety and escalated aggression promoted by alcohol. Modulation of CRF1 function seems to exert a less prominent role over low to moderate alcohol intake, or to species-typical behaviors. While CRF mechanisms in the hypothalamic–pituitary–adrenal axis have some contribution to the neurobiology of alcohol abuse and dependence, a pivotal role for extra-hypothalamic CRF pathways, particularly in the extended amygdala, is well characterized. More recent studies further suggest a direct modulation of brain reward function by CRF signaling in the ventral tegmental area, nucleus accumbens, and the prefrontal cortex, among other structures. This review will further discuss a putative role for other components of the CRF system that contribute for the overall balance of CRF function in reward and stress pathways, including CRF2 receptors, CRF-binding protein, and urocortins, a family of CRF-related peptides.

## Overview

For a few decades now, a critical role for stress in the induction, maintenance, and relapse to drug and alcohol dependence has been increasingly investigated. Indeed, drug dependence has been hypothesized as a “stress surfeit disorder” (1). Dysregulation of brain stress systems would contribute to the transition from drug-taking, which is primarily motivated by reward-seeking, toward escalated drug-taking that becomes mainly instigated by dysphoria and negative reinforcement. As the very primary player mediating brain stress responses, the corticotropin-releasing factor [CRF; also known as corticotropin-releasing hormone (CRH)] has received great attention in drug dependence. This review will discuss evidence provided by preclinical studies focusing on the critical participation of CRF and its closely related peptides urocortins, in escalated alcohol drinking, and other important alcohol-related behaviors. Also, apart from its role in more traditional brain stress circuits, this review will discuss increasing evidence for the CRF system as a direct modulator of brain reward pathways, suggesting that CRF/urocortin signaling may also modulate alcohol’s effects at earlier stages of alcohol dependence. The impact of alcohol exposure on CRF/urocortin signaling and plasticity will be discussed, as potential neuroadaptive mechanisms recruited during the establishment of alcohol dependence. Pharmacological manipulations targeting CRF receptors are presented as promising tools for reducing excessive alcohol drinking, withdrawal-related anxiety as well as alcohol-induced aggression and behavioral sensitization induced by alcohol. The participation of the hypothalamic–pituitary–adrenal (HPA) stress axis is addressed at specific points, but specifically as a downstream target engaged by brain CRF [for reviews on HPA axis and alcohol, refer to Stephens and Wand (2) and Edwards et al. (3)]. Finally, considerations are made on a few human alcohol studies evaluating polymorphisms in CRF-related genes. This review does not attempt to discuss alcohol studies using genetically modified animals for CRF-related genes, a subject vastly covered by a recent review on CRF systems and alcohol (4).

We refer to several review articles published on CRF and drug/alcohol dependence [e.g., Ref. (5–7)] as well as a recent review specifically focusing on CRF mechanisms involved in alcohol use disorders (AUDs) and alcohol-induced neuroplasticity (4).

## Alcohol and Alcohol Use Disorders

Alcohol is the most widely consumed psychotropic substance worldwide. Likewise, the harmful use of alcohol ranks within the five main risk factors for disease, disability, and death in the globe (8). AUDs are responsible for ~6% of all deaths throughout the world, even considering potential beneficial effects of low-risk alcohol drinking (8). Consequences of alcohol misuse can be both acute (e.g., acute intoxication, heavy episodic drinking, driving under the influence of alcohol, alcohol-related violence) and chronic (e.g., AUDs, gastrointestinal diseases including liver cirrhosis, cancers), and cause harm to the drinker, to other individuals, and to society in general. Thus, alcohol consumption is responsible for a large burden in public health, with serious economic and social impact.

Alcohol-related health harm is proportional to the amount of alcohol consumed, with a dose–response relationship. About 13% of the alcohol-drinking population will show a pattern of heavy episodic drinking, or “binge” drinking, which is more commonly associated with alcohol-related injury, including driving accidents and violent behavior (9). AUDs are the main neuropsychiatric condition promoted by alcohol consumption and encompass both the harmful use of alcohol and alcohol dependence. Alcohol dependence affects ~5% of the adult (15 years and older) population worldwide (8) and consists of a variety of behavioral, cognitive, and physiological symptoms that follow repeated alcohol use. According to the International Classification of Diseases, main features of alcohol dependence include a strong desire to consume alcohol; difficulties in controlling the frequency and amount of alcohol consumption; persistent use despite perceived harmful consequences; a higher priority given to obtaining and using alcohol relative to other activities and obligations; increased tolerance; and sometimes a physiological withdrawal state (10).

Despite the complexity of biological, environmental, and social factors that influence drug and AUDs, animal models have been instrumental in providing evidence for behavioral, pharmacological, and neurobiological mechanisms underlying acute and repeated alcohol exposure [for a review, see Ref. (11)]. This review will focus primarily on findings stemming from preclinical studies and how they contribute to our understanding of CRF/urocortin mechanisms in alcohol dependence. Of particular interest are models of voluntary alcohol consumption, including home cage drinking (e.g., two-bottle choice, with intermittent, limited, or continuous access conditions; the “drinking in the dark” model, etc.) and operant self-administration (animals respond by pressing a lever or nose poking in order to obtain an alcohol reward). Using these experimental models, it is also possible to promote escalated, excessive alcohol intake by using protocols of repeated and prolonged voluntary access to the substance, usually followed by one or multiple cycles of withdrawal. Examples are procedures such as the alcohol-deprivation effect (12) and dependent-like alcohol consumption in subsets of chronically drinking rats and mice [e.g., Ref. (13, 14)]. Other widely used procedures for escalated drinking rely on forced alcohol exposure in order to obtain reliable and consistent exposure to high alcohol concentrations, such as the alcohol vapor chamber (15, 16), as well as alcohol-containing liquid diet [see also Ref. (17) for a review]. Such excessive alcohol exposure is usually associated with withdrawal symptoms upon short-term withdrawal, and dysphoria and anxiety during protracted abstinence. These negative features trigger and motivate increased levels of voluntary alcohol self-administration [e.g., Ref. (18); see review by Heilig and Koob (5)].

Different from other drugs of abuse, such as cocaine, amphetamines, and morphine, pharmacological mechanisms for alcohol include a variety of targets in the central nervous system. Synaptic actions of alcohol have been described with several neurotransmitter-gated ionotropic receptors (e.g., GABAA, glutamate NMDA and AMPA receptors, serotonin 5-HT3 receptors, etc.), ion channels (e.g., different subtypes of calcium and potassium channels), as well as intracellular downstream signaling proteins also involved in metabotropic receptors cascades (e.g., diacylglycerol, protein kinase A, protein kinase C, etc.), as extensively reviewed by Lovinger and Roberto (19). Additionally, presynaptic effects of alcohol may be observed, with a facilitation or potentiation of presynaptic GABA release, but not glutamate, as revealed by electrophysiological studies using slice preparations or isolated neurons [reviewed by Lovinger and Roberto (19) and Siggins et al. (20)]. Chronic alcohol exposure produces consistent neuroadaptive changes in the function of both ionotropic and metabotropic glutamate receptors, for example, with the upregulation of NMDA receptors [e.g., Ref. (21–25)]. Likewise, GABAA receptors are markedly affected in their subunit composition, sensitivity, and function by chronic alcohol treatment [see reviews by Grobin et al. (26) and Kumar et al. (27)]. These and many other changes in synaptic function, which vary in different brain regions, are considered critical for the development of tolerance and dependence to alcohol (19).

The effects of alcohol are importantly modulated by neuropeptides, including opioid peptides, neuropeptide Y, orphanin/nociceptin, and orexin. This review will focus on the family of neuropeptides comprised of CRF and its closely related peptides, the urocortins. The next section will briefly describe the physiology of CRF/urocortin signaling in the brain, and subsequently we will discuss the critical role of CRF signaling in AUDs.

## A Glance at CRF/Urocortin Systems in the Brain

The 41-amino acid neuropeptide CRF was identified in 1981 by Vale and colleagues and has long been associated with neural, endocrine, autonomic, and immune responses to stress (28). Acting as a neuromodulator, the availability of CRF is critically determined by the CRF-binding protein (CRF-BP), a glycoprotein that regulates the extracellular availability of CRF to bind to its receptors (29, 30). CRF exerts its effects *via* interaction with two G-protein coupled receptors, namely CRFR1 and CRFR2 (CRF receptor 1 and 2, respectively), which are found in several variants (31). However, CRF has a 10-fold higher affinity for CRFR1 relative to CRFR2 (32). Urocortins, another set of peptides in the CRF family, are the main endogenous ligands for CRFR2, showing similar affinity for both receptor subtypes (urocortin 1), or binding almost exclusively to CRFR2 [urocortins 2 and 3; (33)]. Activation of both CRFR1 and CRFR2 preferentially lead to the activation of cyclic-AMP second messenger pathways (34). The two receptor subtypes are differentially distributed in the brain, with overlapping regions (35). CRFR1 are more ubiquitously found. In rodents, high densities of CRFR1 are found in the anterior hypophysis, cerebral cortex, cerebellum, amygdala, hippocampus, and striatum (36), whereas CRFR2 are more limited to the mesencephalon, raphé nuclei, lateral septum, amygdala, and hypothalamus (37), as recently reviewed by Phillips et al. (4). Although not the focus of the current review, CRF and urocortin peptides, as well as receptors and binding protein, are widely distributed in peripheric organs and tissues, including the gastrointestinal tract, cardiovascular, and immune systems, where they integrate systemic stress responses and participate in other functions [see reviews by Fekete and Zorrilla (33), Pan and Kastin (38), Stengel and Taché (39)].

As a major modulator of systemic neuroendocrine stress responses, hypothalamic CRF drives the HPA axis, with secondary modulation by the neuropeptide arginine vasopressin [e.g., Ref. (28, 40, 41)]. Produced by parvocellular and magnocellular neurons of the paraventricular nucleus of the hypothalamus (PVN), AVP has a limited capacity to activate the HPA axis by itself (4, 42). However, the neuropeptide AVP seems to increase the effects of CRF on HPA axis by synergistic activation of its V1b receptor in the anterior pituitary (4, 42, 43). Produced by parvocellular neurons of the PVN, CRF is released in the median eminence to reach the anterior hypophysis (or pituitary), where it binds to densely expressed receptors, CRFR1 (44). Adrenocorticotropic hormone (ACTH) is then secreted into the blood stream by the adenohypophysis (45). Once secreted, ACTH stimulates the production and release of glucocorticoids from the cortex of adrenal glands (cortisol in humans, corticosterone in rodents). Glucocorticoids will then act upon high- and low-affinity receptors (mineralocorticoid and glucocorticoid receptors, respectively), widely distributed in the periphery and the brain. Cortisol and corticosterone promote adaptive responses to environmental challenges and stressors, including changes in energy metabolism, physiological, and behavioral responses. HPA axis function is importantly regulated *via* inhibitory feedback by glucocorticoids, ultimately reducing activity of PVN neurons and CRF release. For a more complete discussion on HPA axis signaling and functions, we refer to reviews by Herman et al. (45), McEwen (46), Myers et al. (47), Sapolsky et al. (48), and Ulrich-Lai and Herman (49).

Wide distribution of cell bodies and fibers with immunoreactivity for CRF is found in stress-related pathways involving amygdalar nuclei [especially the central nucleus of the amygdala (CeA)], the bed nucleus of the stria terminalis (BNST), and the PVN, with ascending projections to forebrain structures as well as descending innervations to the brain stem (31). High levels of CRF are also detected in the hippocampus, thalamus, locus coeruleus, raphé nuclei, and other mesencephalic structures (50, 51). Detection of neurons and fibers containing urocortin peptides are limited to fewer brain structures and projection sites in rodents, although a wider distribution of brain urocortin can be found in human and non-human primates (38, 52, 53). As extensively reviewed by Fekete and Zorrilla (33), urocortin 1 (Ucn1) is primarily synthesized in the centrally projecting Edinger–Westphal nucleus, a midbrain structure, and other secondary sites. Descending fibers of Ucn1 are found in many regions, including the substantia nigra, the dorsal raphe nucleus, and periaqueductal gray, while ascending fibers of Ucn1 are found in the lateral septum, BNST, hypothalamus, and other structures. Urocortin 2 (Ucn2) is synthesized in the PVN and other hypothalamic nuclei, as well as in the locus coeruleus, but its projection targets are unknown (33). Urocortin 3 (Ucn3) is produced in hypothalamic and amygdala regions. Ucn3 fibers project from the amygdala to the hypothalamic ventral premammillary nucleus, while fibers from undertermined origins are also found in other hypothalamic regions, in lateral septum, BNST, medial amygdala, and ventral hippocampus [for reviews, see Ref. (33, 54)].

Briefly, biological functions mediated by CRF and urocortin peptides include not only stress responses but also regulation of appetitive responses, such as feeding and exploratory behaviors, and a variety of social behaviors (38, 55, 56). In several of these functions, CRF receptor subtypes often play different roles (57, 58). For example, stress and anxiety-like responses are thought to be primarily initiated by CRF/urocortin 1 activation of CRFR1 signaling, while CRFR2 activation has been associated with anxiolytic effects and/or termination of stress responses (38, 59). As another example, CRF and urocortins inhibit feeding behavior likely *via* CRFR2 signaling, although both CRFR1 and CRFR2 are involved in stress-induced anorexigenic effects (54). Thus, biological responses involving CRF and urocortins are wide and complex, and recruitment of specific CRF receptors will vary according to each particular behavior and/or pathology, in a brain region-dependent manner.

Through actions on CRFR1 and/or CRFR2, CRF and urocortin peptides set the pace for brain monoaminergic function in regions, such as the locus coeruleus (primary site for noradrenergic neurons in the brain), the dorsal and median raphé (serotonin neurons), and the ventral tegmental area (VTA), where reward-related dopamine neurons are located [e.g., Ref. (57, 60–62)]. Of particular interest to this review, dopamine neurons in the VTA receive CRF inputs from fibers that originate in the BNST, the CeA and, to a lesser extent, the hypothalamic PVN (63). In the VTA, CRF stimulates firing of dopamine neurons likely *via* CRFR1 signaling, modulating dopamine output to the nucleus accumbens (64). On the other hand, when CRF is directly applied in the nucleus accumbens, it can increase dopamine release and promote appetitive behavior (65). Exposure to acute stress is well known to induce activation of this dopamine pathway arising from the VTA and projecting to the nucleus accumbens and the prefrontal cortex, typically promoting increased drug-taking and/or drug-seeking behaviors [see review by Holly and Miczek (66)]. Interestingly, the ability of stress to trigger activation of dopamine brain reward pathways may be a downstream event from stress-induced increased CRF signaling within the VTA. For example, acute exposure to footshock stress promotes marked increases in CRF levels in the VTA and is associated with dopamine increases in the VTA and with reinstatement of cocaine-seeking behavior in cocaine-experienced rats (67). Remarkably, these CRF effects seem to be primarily mediated by CRFR2 in the VTA (68). More recently, acute or repeated social defeat stress were also shown to phasically increase CRF levels in the VTA, but with regional heterogeneity (anterior vs. posterior subregions of VTA), while repeated stress promotes long-term increases in CRF tonus in both subregions (69). In this case, both CRFR1 and CRFR2 modulated cocaine-seeking behaviors in rats with a history of social stress, according to the subregion of the VTA (69). Evidence for the involvement of CRF/urocortin signaling in alcohol drinking and other alcohol-related behaviors will be specifically addressed in the following sections.

## Modulation of Alcohol Consumption by Pharmacological Manipulations of CRF/Urocortin Signaling

Extensive evidence points to a critical role for CRF and CRFR1 receptors in escalated alcohol consumption [(70–74); see review by Phillips et al. (4)]. As summarized in Table 1, antalarmin – a non-peptidergic CRFR1 antagonist – reduces free-choice alcohol drinking in rats given intermittent access to alcohol (72). Mice chronically exposed to alcohol vapor showed reduced escalated alcohol self-administration when treated with antalarmin prior to the withdrawal drinking session (75). In this study, control mice showed no changes in alcohol self-administration when treated with antalarmin, suggesting that only excessive drinking, in this case withdrawal-induced drinking, was sensitive to CRFR1 treatment (75). Binge drinking in C57BL6 mice was attenuated by another CRFR1 antagonist, CP-154,526, using the drinking-in-the-dark protocol (76). However, alcohol consumption was only reduced in high drinking conditions (blood alcohol ~80 mg/dl), not in moderate drinking conditions (blood alcohol ~40 mg/dl or lower). Other systemically administered CRFR1 antagonists were also effective in attenuating drinking-in-the-dark consumption, such as CP 376,395 and NBI-27914, despite non-selective effects reducing water and calory intake as well (77). The possibility that reduced alcohol drinking due to CRFR1 blockade could be secondary to broader effects on fluid and calory intake needs to be further investigated (77). A couple of studies failed to observe an attenuation of alcohol intake by CRFR1 antagonist when animals had continuous access to the drug [e.g., Ref. (78, 79)].

**Table 1:** **Summarized effects of pharmacological manipulations of CRF/urocortin targets on alcohol-drinking studies**.

Receptor	Mechanism	Drug	Drug administration	Results	Reference
CRFR1	Antagonist	Antalarmin	ip	Reduced escalated^a^ drinking	Lodge and Lawrence (71), Cippitelli et al. (72), Hansson et al. (70), Chu et al. (75), Marinelli et al. (80), Lowery-Gionta et al. (73)
				Reduced ethanol seeking	Marinelli et al. (80), Funk et al. (81)
				No effect on escalated^a^ drinking	Yang et al. (79), Molander et al. (78)
			Into CeA	Reduced escalated^a^ drinking	Lowery-Gionta et al. (73)
CRFR1	Antagonist	CP-154,526	ip	Reduced stress-induced reinstatement of ethanol seeking	Le et al. (82)
				Reduced escalated^a^ drinking	Correia et al. (83), Hwa et al. (84), Lowery et al. (85, 86), Overstreet et al. (87), Sparta et al. (76, 88)
			icv (*chronic minipump*)	No effect on escalated^a^ drinking	Hwa et al. (84)
			Into VTA	Reduced escalated^a^ drinking	Hwa et al. (74), Sparta et al. (89)
			Into DRN	Reduced escalated^a^ drinking	Hwa et al. (74, 84)
			Into MRN	No effect on escalated^a^ drinking	Hwa et al. (84)
CRFR1	Antagonist	CP 376,395	ip	Reduced escalated^a^ drinking	Giardino and Ryabinin (77), Hwa et al. (84), Simms et al. (90)
			icv (*chronic minipump*)	Reduced escalated^a^ drinking only on day 1; no effect in remaining days	Hwa et al. (84)
CRFR1	Antagonist	CRA-1000	ip	Reduced escalated^a^ drinking	Overstreet et al. (87, 91), Lowery-Gionta et al. (73)
CRFR1	Antagonist	LWH-63	ip	Reduced escalated^a^ drinking	Lowery-Gionta et al. (73)
			sc	Reduced escalated^a^ drinking	Sabino et al. (92)
				Modestly increased limited access alcohol and water drinking	Sabino et al. (92)
CRFR1	Antagonist	MJL-1-109-2	ip	Reduced escalated^a^ drinking	Funk et al. (81)
				No effect on drinking	Sabino et al. (92)
CRFR1	Antagonist	MPZP	sc	Reduced escalated^a^ drinking	Gilpin et al. (93), Richardson et al. (94)
				No effect on escalated^a^ drinking	Ji et al. (95)
CRFR1	Antagonist	MTIP	ip	Reduced stress-induced reinstatement of ethanol seeking	Gehlert et al. (96)
				Reduced escalated^a^ drinking	Gehlert et al. (96)
CRFR1	Antagonist	NBI-27914	ip	Reduced escalated^a^ drinking	Lowery-Gionta et al. (73), Giardino and Ryabinin (77)
CRFR1	Antagonist	NBI-27914	ip	No effect on escalated^a^ drinking	Molander et al. (78)
CRFR1	Antagonist	R121919	ip	No effect on escalated^a^ drinking	Yang et al. (79)
			sc	Increased escalated^a^ drinking	Sabino et al. (92)
				Prevented stress-induced suppression of drinking	Sabino et al. (92)
				No effect on escalated^a^ drinking	Sabino et al. (97)
				Reduced escalated^a^ drinking	Funk et al. (81), Roberto et al. (24), Roltsch et al. (98)
CRFR1	Antagonist	SSR125543	Into NAcc	Reduced escalated^a^ drinking	Knapp et al. (99)
			Into AMY, DRN	No effect on escalated^a^ drinking	Knapp et al. (99)
CRFR1/2	Agonist	CRF	icv	No effect on escalated^a^ drinking	O’Callaghan et al. (100)
				Reduced escalated^a^ drinking	Bell et al. (101), Thorsell et al. (102)
				Reinstated CRF-induced ethanol seeking	Le et al. (103)
			Into DRN	No effect on drinking	Weitemier and Ryabinin (104)
			Into LS	Reduced escalated^a^ drinking and water intake	Ryabinin et al. (105)
			Into MRN	Reinstated CRF-induced ethanol seeking	Le et al. (103)
			Into NAcc	Further augmented escalated^a^ drinking	Knapp et al. (99)
CRFR1/2	Agonist	CRF	Into CeA, DRN, VTA, PVN	No effect on escalated^a^ drinking	Knapp et al. (99)
CRFR1/2	Agonist	Ucn 1	Into DRN	No effect on drinking and reduced water intake	Weitemier and Ryabinin (104)
			Into LS	Reduced escalated^a^ drinking	Ryabinin et al. (105)
CRFR1/2	Antagonist	d-Phe-CRF	icv	Reduced escalated^a^ drinking	Valdez et al. (106)
				Reduced reinstatement of ethanol seeking induced by the combination of stress and ethanol-cues	Liu and Weiss (107)
				Reduced stress-induced reinstatement of ethanol seeking	Le et al. (82), Liu and Weiss (107)
				No effect on cue-induced reinstatement of ethanol seeking	Liu and Weiss (107)
			Into CeA	Reduced escalated^a^ drinking	Finn et al. (108), Funk et al. (109, 110)
			Into MRN	Reduced yohimbine-induced reinstatement	Le et al. (111)
			Into MRN	Reduced stress-induced reinstatement	Le et al. (103)
CRFR1/2	Antagonist	Alpha-helical CRF	icv	Increased drinking in low preference animals	O’Callaghan et al. (100)
				Reduced escalated^a^ drinking	Lowery et al. (86)
				No effect on drinking in high preference animals	O’Callaghan et al. (100)
CRFR2	Agonist	Ucn 3	icv	Reduced escalated^a^ drinking	Sharpe and Phillips (112), Valdez et al. (113), Lowery et al. (86)
			Into CeA	Reduced escalated^a^ drinking	Funk and Koob (114)
				Increased drinking in control animals	Funk and Koob (114)
CRFR2	Antagonist	Astressin-2B	Into VTA	Reduced escalated^a^ drinking	Albrechet-Souza et al. (115)
			Into CeA	No effect on escalated^a^ drinking	Albrechet-Souza et al. (115)
CRFR2	Antagonist	Antisauvagine-30	Into DRN	No effect on drinking	Weitemier and Ryabinin (104)
CRF-BP	Antagonist	CRF_(6–33)_	Into VTA	Reduced escalated^a^ drinking	Albrechet-Souza et al. (115)
			Into CeA	No effect on escalated^a^ drinking	Albrechet-Souza et al. (115)

*^a^*Escalated drinking* refers to ethanol intake that is above baseline or control levels, as observed in protocols of alcohol deprivation, “binge” drinking (as in drinking-in-the-dark protocol), intermittent/limited access conditions, withdrawal from ethanol vapor or liquid diet exposure, prolonged ethanol access conditions, etc*.

*ip, intraperitoneal; sc, subcutaneous; icv, intracerebroventricular; AMY, amygdala; CeA, central nucleus of the amygdala; BLA, basolateral amygdala; DRN, dorsal raphé nucleus; MRN, median raphé nucleus; PVN, paraventricular nucleus of the hypothalamus; VTA, ventral tegmental area; NAcc, nucleus accumbens; LS, lateral septum*.

Overall, these studies suggest that systemic CRFR1 antagonists present selective actions in reducing escalated alcohol drinking, but not moderate drinking, as summarized in Table 1 [e.g., Ref. (75, 76, 88)]. One particular study suggests that these effects are likely extra-hypothalamic and independent from HPA axis activation, since adrenalectomized mice show similar binge alcohol intake as controls, and CRFR1 blockade remains effective in attenuating drinking in adrenalectomized animals (86). Non-selective peptidergic CRF receptor antagonists, such as alpha-helical CRF or d-Phe-CRF, usually present a similar profile of effects as those of selective CRFR1 antagonists. In particular, icv infusion of d-Phe-CRF has been shown to reduce escalated alcohol drinking (106) and prevent reinstatement of stress-induced alcohol seeking (82, 107), just like more selective CRFR1 antagonists (see Table 1). When a selective CRFR1 antagonist was given chronically *via* icv minipumps, however, reduced drinking was only observed on the first drinking day and not on the following test sessions, suggesting that the chronic, continuous blockade of CRFR1 may not be as effective (84).

Important brain regions for the anti-drinking effects of selective CRFR1 and non-selective CRFR1/2 antagonists include brain reward and stress-related regions. For example, infusion of CP-154,526 into the VTA attenuated binge drinking in C57BL/6J mice (89). A different study also showed a reduction in two-bottle choice drinking in both rats and mice after intra-VTA infusion of CP-154,526 (74). CRFR1 signaling in the dorsal raphé nucleus (DRN) is also recruited for escalated alcohol drinking in rats and mice, likely due to a CRFR1 modulation of serotonergic output to the prefrontal cortex (74, 84). On the other hand, while CRFR1 in the median raphé nucleus may not modulate drinking (84), blockade of CRFR1/2 in this region prevents stress-induced reinstatement of alcohol seeking (103, 111). Infusion of either the non-selective antagonist d-Phe-CRF or antalarmin into the CeA also attenuates binge drinking (73) and withdrawal-induced alcohol self-administration (108–110). Notably, the preferential, but non-selective, activation of CRFR1 by CRF itself, whether icv or in different brain regions, produces inconsistent outcomes in alcohol drinking [e.g., Ref. (100, 101, 104, 105)]. Nonetheless, infusion of CRF into the ventricles or into the median raphé promotes reinstatement of alcohol-seeking behavior (103).

Using a different procedure, high alcohol-preferring P rats were exposed to three cycles of voluntary alcohol drinking (5 days), with 2-day withdrawal periods in between cycles (99). During withdrawal periods, rats received the infusion of a CRFR1 antagonist, SSR 125543, into the nucleus accumbens prior to exposure to restraint stress. Escalated drinking was promoted by the multiple cycles of withdrawal and stress exposure, and was attenuated by the blockade of CRFR1 in the accumbens during withdrawal periods (99). Using the same protocol, CRFR1 blockade in the DRN or the amygdala failed to affect escalated alcohol drinking (99). Interestingly, intra-accumbens infusion of CRF itself during the withdrawal periods (as a substitute for restraint stress) augmented alcohol consumption but had no effects on drinking when microinjected into the VTA, amygdala, DRN, or the paraventricular nucleus of the hypothalamus (99). Thus, plastic changes in accumbal CRFR1 seem to be recruited during cycles of withdrawal and stress, which underlie escalated drinking.

A few studies also suggest the involvement of urocortin peptides and CRFR2 in the modulation of escalated alcohol drinking. In mice, infusion of Ucn 1 (a non-selective agonist at CRFR1/2) into the lateral septum, but not into the dorsal raphé, significantly blunts binge alcohol drinking, likely due to a preferential action on CRFR2 (105). The selective activation of CRFR2 with icv administration of Ucn 3 reduced alcohol intake in mice (86, 112) and rats (113). Interestingly, this attenuation of escalated alcohol consumption can also be observed after infusion of Ucn 3 into the CeA of rats withdrawn from chronic alcohol vapor (114). On the other hand, blockade of CRFR2 in the CeA with astressin-2B failed to affect binge alcohol drinking in mice (115). Thus, these studies suggest that it is the activation, not the blockade of CRFR2, which prevents or attenuates excessive alcohol drinking, icv, or in regions such as the lateral septum or the CeA. However, one recent study reported decreased alcohol intake after infusion of a selective CRFR2 antagonist into the VTA (115), suggesting that different roles for CRFR2 may emerge from different brain pathways.

In summary, both subtypes of CRF receptors seem to be important during escalated alcohol drinking. While consistent and extensive evidence weighs toward CRFR1 modulation of excessive alcohol intake, increasing studies are currently suggesting that a balance between CRFR1 and CRFR2 activation/blockade may be critical in determining the final outcome. In particular brain regions such as the CeA, it seems that the inhibition of CRFR1 signaling and/or facilitation of CRFR2 function will blunt excessive alcohol drinking. On the other hand, blockade of either CRFR1 or CRFR2 in the VTA is effective in attenuating drinking (74, 89, 115). Thus, both CRFR1 and CRFR2 are important pharmacological targets that can contribute to treatment of AUDs. Furthermore, targets, such as the CRF-BP, have just started to emerge as interesting modulators of alcohol drinking, as recently suggested by Albrechet-Souza et al. (115).

## CRF/Urocortin Modulation of Other Alcohol-Related Behaviors

### Conditioned Place Preference

Conditioned place preference is a common model for indirectly assessing drug reward and drug seeking, by associating drug-induced reinforcing effects to a particular environment [for reviews, see Ref. (116–118)]. In this model, alcohol has reliably produced conditioned reward as evidenced by increased time spent in the alcohol-paired environment, relative to a different saline-paired environment, when the animal is tested in the absence of the drug (i.e., conditioned preference).

Genetic manipulations of the CRF system seem to affect the conditioned reinforcing effects of alcohol. Olive et al. (119) showed that transgenic CRF knockout mice fail to present preference to the alcohol-paired environment after conditioning sessions with a moderate dose of alcohol (2 g/kg), in a protocol that promotes significant preference in wild-type controls. Nonetheless, with a higher conditioning dose of alcohol (3 g/kg), both wild-type and knockout mice presented conditioned preference for the alcohol-associated context (119). These results suggest that CRF may have a facilitatory role in alcohol-conditioned reward, but other mechanisms contribute as well.

Urocortin1 knockout mice failed to present preference to the alcohol-paired environment (2 g/kg conditioning dose), and this strain also presents decreased alcohol intake and preference in a two-bottle choice protocol (120). Nonetheless, alcohol-conditioned aversive effects were preserved in this strain, suggesting that different mechanisms underlie conditioned reward and conditioned aversion (120). Furthermore, CRFR2 knockout mice do not display alcohol-induced conditioned place preference (120). Since Ucn1 acts on both CRFR1 and CRFR2, authors suggest that Ucn1-mediated activation of CRFR2 would be critical for the conditioned effects of alcohol. These were the only two studies assessing conditioned place preference for alcohol and CRF/urocortin systems. Determination of specific mechanisms requires assessment of the role of CRFR1 in this model, and further pharmacological studies.

### Behavioral Sensitization

Similar to other drugs of abuse, repeated injections of alcohol promotes neuroadaptation in brain reward pathways, rendering the brain more vulnerable and sensitive to drug-induced reward and stimulation [see reviews by Robinson and Berridge (121, 122), Steketee and Kalivas (123), Vanderschuren and Pierce (124)]. Behaviorally, this neurochemical sensitization may be accompanied by progressively augmented motor stimulation responses or “behavioral sensitization” [e.g., Ref. (125–129)]. Different from other drugs, however, alcohol-induced locomotor sensitization is not easily demonstrated in rats^1^ (130, 131) but is well described in some inbred and outbred strains of mice [e.g., Ref. (132–135); see also Ref. (136) for a report on adolescent macaques]. Also, a putative role for sensitized behavioral responses to alcohol has been suggested as a vulnerability factor for alcohol dependence in humans (137, 138).

Components of the CRF system play a critical role in alcohol-induced behavioral sensitization, as also reviewed by Phillips et al. (4). Studies with transgenic mice show that CRF and CRFR1 are required for alcohol sensitization (139, 140). Mice homozygous for CRF gene depletion do not show a sensitized locomotor response to alcohol after repeated injections, while heterozygous animals develop normal levels of sensitization (140). CRFR1 knockout mice failed to develop alcohol sensitization and also show a reduction in the acute response to alcohol (119, 139). On the other hand, knockout mouse lines for CRFR2 or urocortin 1 both develop normal levels of sensitization (139), suggesting no particular role for CRFR2 in alcohol sensitization.

As shown in Table 2, pharmacological blockade of CRFR1 receptors with CP-154,526 (in doses of 10 mg/kg and higher; ip) consistently abolishes the expression of a sensitized locomotor stimulation after a 10-day alcohol treatment regimen, without affecting the acute response to alcohol in DBA/2J mice (139, 141). During the induction phase, the effects of the CRFR1 antagonist are less clear, with significant attenuation of alcohol sensitization only at a high 30 mg/kg dose of CP-154,526 [no effects at lower doses; (139, 141)]. No studies were found assessing the pharmacological manipulation of CRFR1 in different inbred or outbred lines of mice other than DBA/2J, nor were these CRFR1 pharmacological manipulations tested in particular brain sites, as shown in Table 2. Future studies should broaden our understanding of the critical CRF/CRFR1 mechanisms underlying alcohol sensitization. Furthermore, while glucocorticoids seem to play a role in alcohol sensitization (139, 140, 142–144), it remains to be determined whether glucocorticoid involvement in this process is directly mediated by upstream CRF signaling.

**Table 2:** **Pharmacological manipulations of CRF/urocortin targets on alcohol-related behaviors**.

Target	Drug (action, route adm.)	Experimental design/treatment	Results	Reference
**Behavioral sensitization**				
CRFR1	CP-154,526 (antagonist, ip)	DBA/2J mice. Ethanol injections (2.5 g/kg, ip) for 10 days, followed by ethanol challenge (1.5 g/kg, ip). Pretreatment with antagonist during acquisition or expression of behavioral sensitization	CRFR1 antagonist blocked the expression of ethanol-induced locomotor sensitization when administered prior to the challenge, but not during repeated ethanol treatment (*induction*)	Fee et al. (141)
		DBA/2J mice. Ethanol injections (2.5 g/kg, ip) for 10 days, followed by ethanol challenge (1.5 g/kg, ip). Pretreatment with antagonist during acquisition or expression of behavioral sensitization	CRFR1 antagonist blocked ethanol-induced locomotor sensitization when administered during the induction phase or prior to the challenge (*expression*)	Pastor et al. (139)
**Aggression**				
CRFR1	CP-154,526 or MTIP (antagonist, ip)	CFW Swiss-derived mice. Operant ethanol self-adm (1.0 g/kg, oral, ~twice/week for 6 weeks); antagonist injection immediately after drinking, 10 min before aggressive confrontation	Reduced alcohol-escalated aggression and species-typical aggression	Quadros et al. (145)
	CP-154,526 or MTIP (antagonist, into DRN)	CFW Swiss-derived mice. Operant ethanol self-adm (1.0 g/kg, oral, ~twice/week for 6 weeks); antagonist infusion immediately after drinking, 10 min before aggressive confrontation	Selective reduction of alcohol-escalated aggression, with no effect on species-typical aggressive behaviors	Quadros et al. (145)
**Elevated plus maze**				
CRFR1	MTIP (antagonist, ip)	Wistar and msP rats. Ethanol injection (3 g/kg, ip) administered 12 h before elevated plus maze test. Antagonist administered 30 min prior test	Reduction of anxiogenic effects elicited by acute ethanol withdrawal	Gehlert et al. (96)
CRFR1/2	Alfa-helical CRF (9–41) (antagonist, icv)	Wistar rats. Ethanol liquid diet (2–3 weeks); antagonist administered 8 h into ethanol withdrawal. Anxiety-like behavior tested 30 min after drug adm	Reduction of anxiogenic effects elicited by ethanol withdrawal	Baldwin et al. (146)
		Wistar rats. Ethanol liquid diet (16 days); antagonist administered 8 h into ethanol withdrawal. Anxiety-like behavior tested 30 min after drug adm	No change in the anxiogenic effects induced by ethanol withdrawal	Rassnick et al. (147)
	Alfa-helical CRF (antagonist, into CeA)	Wistar rats. Ethanol liquid diet (16 days); antagonist administered 8 h after ethanol withdrawal. Behavioral test conducted 30 min after drug adm	Reduction of anxiogenic effects elicited by ethanol withdrawal	Rassnick et al. (147)
	d-Phe-CRF (antagonist, icv)	Wistar rats. Ethanol liquid diet (21 days) + 6 weeks withdrawal; antagonist administered prior to restraint stress (15 min of stress). Elevated plus maze test was conducted after exposure to the stressor	Conditions of protracted abstinence and restraint stress, *per se*, failed to affect anxiety-like behavior. When combined, restraint stress-induced anxiogenic effects in animals preexposed to ethanol, which was attenuated by the antagonist	Valdez et al. (148)
CRFR2	Ucn 3 (agonist, icv)	Wistar rats. Operant self-adm (daily 30-min session, 22 days) followed by ethanol liquid diet (21 days). Agonist administered 2 h into ethanol withdrawal. Elevated plus maze was conducted 10 min after drug adm	Reduction of anxiogenic effects elicited by ethanol withdrawal	Valdez et al. (113)
**Social interaction**				
CRFR1	CP-154,526 (antagonist, ip)	Sprague-Dawley rats. Ethanol liquid diet (3 weeks, 5 days/week); antagonist administered 4 h after ethanol removal, during first two withdrawal periods. Restraint stress (45 min) was applied 3 days after the final ethanol withdrawal. Social interaction test 30 min after stress	The antagonist reduced social avoidance promoted by ethanol withdrawal in combination with an acute stressor	Breese et al. (149)
		Sprague-Dawley rats. Ethanol liquid diet (3 weeks, 5 days/week); antagonist administered 4 h after ethanol removal, during first two withdrawal periods. Social interaction test 5–48 h after final ethanol withdrawal	5-h into ethanol withdrawal, social avoidance was reduced by the antagonist. Neither 24 or 48 h of withdrawal produced social deficits	Wills et al. (150)
		Sprague-Dawley rats. Ethanol liquid diet (3 weeks, 5 days/week); antagonist administered 4 h after ethanol removal, during first two withdrawal periods. Social interaction test conducted 5 h after the final ethanol withdrawal	The antagonist blocked social avoidance induced by ethanol withdrawal	Overstreet et al. (91)
CRFR1	CP-154,526 (antagonist, ip)	P rats. Ethanol liquid diet (3 weeks, 5 days/week); antagonist administered 4 h after ethanol removal, during first two withdrawal periods. Social interaction test conducted 5–6 h after the final ethanol withdrawal	Antagonist blocked ethanol-withdrawal-induced social anxiety in P rats	Overstreet et al. (151)
CRFR1	CRA-(1000) (antagonist, ip)	Sprague-Dawley rats. Control liquid diet (11 days) with two non-consecutive days of restraint stress (60 min; days 6 and 11). Antagonist administered 30 min before restraint stress. Starting on day 12, animals received ethanol liquid diet (5 days). Social interaction tested 5 h into ethanol withdrawal	The antagonist prevented the facilitatory effect of stress on ethanol-withdrawal anxiety	Breese et al. (152)
		Sprague-Dawley rats. Ethanol liquid diet (17 days); drug administered 30 min before social interaction test (5–6 h into ethanol withdrawal)	The antagonist blocked ethanol-withdrawal-induced social anxiety	Knapp et al. (153)
		Sprague-Dawley rats. Ethanol liquid diet (3 weeks, 5 days/week); antagonist administered 4 h into ethanol withdrawal, during the first two withdrawal periods, or 30 min before social interaction test (5 h into final ethanol withdrawal)	The antagonist reduced ethanol-withdrawal-induced social anxiety, when administered during the first withdrawal periods (preventing withdrawal sensitization), or 30 min prior to the social interaction test	Overstreet et al. (91)
CRFR1	SSR 125543 (antagonist, into CeA, DRN, or dorsal BNST)	Sprague-Dawley rats. Control liquid diet (12 days) with two non-consecutive days of restraint stress (60 min; days 6 and 12). Antagonist infused 15 min before restraint stress. Then, exposure to ethanol liquid diet (5 days). Social interaction test 5–6 h into ethanol withdrawal	Infusion of a CRFR1 antagonist into CeA, DRN, and dorsal BNST prevented the stress-potentiation of ethanol-withdrawal anxiety	Huang et al. (154)
	SSR 125543 (antagonist, into DRN, amygdala, or NAcc)	Inbred alcohol-preferring (iP) rats. Single-bottle continuous access (3 days), then two-bottle choice continuous access (3 weeks; 5 days/week). Antagonist administered 15 min before restraint stress (60 min), which occurred 4 h into ethanol withdrawal, during the first two withdrawal periods. Social interaction 5–6 h after the final ethanol withdrawal	Infusion of the antagonist into DRN and amygdala prevented social avoidance induced by the combination of ethanol withdrawal and restraint stress. No effects of the antagonist when infused into the NAcc	Knapp et al. (99)
	SSR 125543 (antagonist, ip)	Sprague-Dawley rats. Control liquid diet (13 days) with two non-consecutive days of cytokine or chemokine treatment (days 7 and 13). Antagonist administered 15 min before treatment. Then, animals received ethanol liquid diet (5 days). Social interaction tested 24 h into ethanol withdrawal	Pretreatment with cytokine or chemokine elicited social anxiety after withdrawal from short-term exposure to ethanol. Social avoidance was prevented by the CRFR1 antagonist given during pretreatment phase	Knapp et al. (155)
CRFR1/2	CRF (agonist, icv)	Sprague-Dawley rats. Control liquid diet with two non-consecutive days of CRF infusion (days 1 and 6). Then, animals received ethanol liquid diet (5 days). Social interaction test was conducted 5 h into ethanol withdrawal	Pretreatment with CRF increased the social anxiety induced by withdrawal from one cycle of ethanol exposure	Overstreet et al. (91)
CRFR1/2	CRF (agonist, into DRN, amygdala, NAcc, VTA, or PVN)	Inbred alcohol-preferring (iP) rats. Single-bottle continuous access (3 days), then two-bottle choice continuous access (3 weeks; 5 days/week). CRF given 4 h into ethanol withdrawal during the first two withdrawal periods. Social interaction test 5–6 h into final ethanol withdrawal	Infusion of CRF into DRN or amygdala increased ethanol withdrawal-induced social anxiety. No effects after CRF infusion into NAcc, VTA, or PVN	Knapp et al. (99)
	CRF (agonist, into CeA, BLA, DRN, dBNST, vBNST, hippocampus CA1, or PVN) and SSR 125543 (CRFR1 antagonist, ip)	Sprague-Dawley rats. Control liquid diet (12 days) with two non-consecutive days of CRF injection on different brain sites (days 6 and 12). In other experiments, CRFR1 antagonist was administered (ip) 15 min before CRF infusion. Then, animals received ethanol liquid diet (5 days). Social interaction test 5–6 h into ethanol withdrawal	Pretreatment with CRF into CeA, DRN, BLA, or dorsal BNST increased ethanol withdrawal-induced social anxiety. No effects when CRF was infused into ventral BNST, CA1, or PVN. The administration of the CRFR1 antagonist prevented the enhanced withdrawal anxiety induced by CRF infusions into CeA, DRN, and dorsal BNST	Huang et al. (154)
CRFR2	Antisauvagine-30 (antagonist, icv)	Sprague-Dawley rats. Ethanol liquid diet (3 weeks, 5 days/week); antagonist administered 4 h into ethanol withdrawal, during the first two withdrawal periods. Social interaction test 5 h into final ethanol withdrawal	No effects in ethanol withdrawal-induced social anxiety	Overstreet et al. (91)
	Ucn 3 (agonist, icv)	Sprague-Dawley rats. Control liquid diet (12 days) with two non-consecutive days of CRF injection (days 6 and 12). Then, animals received ethanol liquid diet (5 days). Social interaction test 5–6 h into ethanol withdrawal	No effects on ethanol withdrawal-induced social anxiety	Huang et al. (154)

*ip, intraperitoneal; icv, intracerebroventricular; CeA, central nucleus of the amygdala; BLA, basolateral amygdala; DNR, dorsal raphe nucleus; PVN, paraventricular nucleus of the hypothalamus; BNST, bed nucleus of the stria terminalis (d, dorsal; v, ventral); VTA, ventral tegmental area; NAcc, nucleus accumbens*.

### Alcohol-Escalated Aggression

Moderate doses of alcohol may promote escalated levels of aggressive behaviors in mice, rats, monkeys, and humans [see reviews from Miczek et al. (156, 157)]. In mice, alcohol-heightened aggression seems to involve serotonin transmission arising from the DRN and involving different serotonin receptors and terminal regions [e.g., Ref. (158–161)]. Interestingly, CRF receptors are well established critical modulators of serotonin function in the dorsal raphé [e.g., Ref. (57, 58, 162), for reviews], and we have observed a critical role for raphé CRFR1 in the modulation of alcohol-related aggression in male mice (145). Systemic administration of two different CRFR1 antagonists (CP-154,526 and MTIP) was shown to dose-dependently reduce aggression not only in mice that were consuming a low dose of alcohol (1.0 g/kg) but also in mice under control conditions (after water drinking). However, when the CRFR1 antagonists were administered intra-dorsal raphé, a selective reduction in alcohol-escalated aggression was reported, with no other effects on species-typical aggressive behavior (145). Furthermore, the anti-aggressive effects of CP-154,526 seem to rely upon serotonin transmission, with increased serotonin output to the prefrontal cortex as a result of CRFR1 antagonism in alcohol-drinking animals (145).

### Alcohol Withdrawal-Induced Effects on Anxiety-Like Behaviors

In models with extensive and prolonged exposure to alcohol (e.g., alcohol vapor chamber or feeding from an alcohol-containing liquid diet), animals may present alcohol withdrawal effects within a few hours or days post termination of alcohol treatment, as well as protracted abstinence signs. Increases in anxiety-like behavior are a well characterized consequence of alcohol withdrawal, particularly within the first hours of withdrawal. Using the elevated plus maze, withdrawal-induced anxiety-like behavior was attenuated by icv administration of the non-selective CRFR1/R2 antagonist, alpha-helical CRF (146). However, a subsequent study only observed anti-anxiety effects when alpha-helical CRF was injected into the CeA, but not icv (147). In protracted abstinence from a 3-week alcohol exposure, anxiogenic effects in the plus maze emerged upon exposure to a stressor, selectively in alcohol “dependent” subjects. This effect was prevented by centrally blocking CRF receptors prior to the stressor (148). Specific roles for CRFR1 or CRFR2 mechanisms in this particular model are still inconclusive. On the one hand, the activation of CRFR2 with urocortin 3 reduced anxiety behavior after withdrawal from chronic alcohol (113). On the other hand, selectively blocking CRFR1 receptors reduced anxiety-like behavior after withdrawal from a single alcohol injection (96).

A greater number of studies addressed social anxiety as an index of alcohol withdrawal, as assessed in social interaction tests, as shown in Table 2. Most commonly, these studies exposed rats to an alcohol liquid diet, with a single or repeated cycles of withdrawal (e.g., one cycle consists of 5 days of alcohol diet, followed by 2 days of alcohol withdrawal). This protocol promotes reduced social interaction when animals are tested a few hours into withdrawal, usually after the third cycle of alcohol exposure (see experimental designs on Table 2). Pharmacological antagonism of CRFR1 consistently attenuates repeated alcohol-withdrawal-induced social anxiety. Different CRFR1 antagonists are effective in reducing social avoidance when administered shortly prior to the social interaction test [e.g., Ref. (91, 149, 153)]. Interestingly, blocking CRFR1 signaling during the initial withdrawal days of the cycles of alcohol exposure/withdrawal also prevents the typical social avoidance response, even though animals are tested in the absence of the antagonist [e.g., Ref. (91, 149–151)]. This suggests that cumulative neuroadaptations involving CRF occur over the repeated cycles of exposure and withdrawal, and by blocking CRFR1 receptors attenuates the deleterious effects of withdrawal on social anxiety. A few brain regions have been shown to participate in the CRFR1 modulation of withdrawal-related anxiety, including the DRN and the CeA, but not the nucleus accumbens (99).

Furthermore, exposure to stressors or to an infusion of CRF may additionally aggravate alcohol withdrawal-related social anxiety with a single cycle of alcohol diet exposure (91, 154), or with a two-bottle choice protocol (99). Infusion of CRF directly into the DRN, CeA, BNST, and the basolateral amygdala (BLA), either prior to alcohol exposure or during withdrawal periods of drinking cycles, also potentiates social avoidance associated with alcohol withdrawal (99). These effects of CRF are likely mediated by CRFR1, since urocortin 3 infusion does not replicate the CRF potentiation of alcohol withdrawal (154), neither does a CRFR2 antagonist block withdrawal-induced social anxiety (91).

## Consequences of Alcohol Exposure on the CRF/Urocortin System

Extensive and consistent evidence supports alcohol’s actions on the CRF systems, modulating both HPA axis function as well as extra-hypothalamic CRF signaling in different brain regions. However, as shown in Table 3, consequences of alcohol exposure on CRF/urocortin system and the HPA axis may vary according to several variables, such as the route of alcohol administration, duration of alcohol exposure, alcohol withdrawal period, age, sex, animal strain, and CRF-related targets. Because the primary interest of this review is focused on alcohol and CRF-related peptides and signaling, data on ACTH and corticosterone were only included from studies that also directly assessed CRF-related targets. For reviews analyzing alcohol’s effects on HPA axis, with focus on glucocorticoid systems, please refer to Rose et al. (163), Stephens and Wand (2), and Edwards et al. (3).

**Table 3:** **Consequences of ethanol exposure on CRF/urocortin systems**.

Target	Tissue/brain region	Ethanol administration	Withdrawal period	Other manipulations	Animal/age	Results	Reference
ACTH	Blood	*ip injection*: acute (3 g/kg)	0–3 h	Astressin (non-selective CRF receptor antagonist; 3 mg/kg iv)	Rats: Sprague-Dawley (male) – adult	Acute ethanol increases ACTH levels (peak at 30 min; baseline restored after 3 h). ACTH response to ethanol is blunted by pretreatment with astressin	Rivier and Lee (164)
ACTH	Blood	*ip injection*: acute 1.5–3 g/kg	0–30 min	Non-selective CRF receptor antagonists: α-helical CRF_9–41_ (25 μg, icv) and astressin (0.3–3 mg/kg, iv)	Rats: Sprague-Dawley (male) – adult	Acute ethanol increased ACTH levels, with peak 15 min after ethanol injection. Pretreatment with astressin attenuated the increased ACTH response to ethanol. Prior injection of α-helical CRF failed to alter ethanol-induced ACTH response	Rivier et al. (165)
ACTH	Pituitary	*Acute ethanol exposure* in hypothalamic–pituitary superfusion or pituitary superfusion (various concentrations)	During acute ethanol incubation		Rats: Sprague-Dawley (male) – adult	Incubation of hypothalamus–pituitary superfusion with acute ethanol produced dose-dependent increases on ACTH release (peak at 20 mg % ethanol). Acute ethanol on pituitary superfusion also produced increased ACTH release (peak at 40 mg % ethanol)	Redei et al. (166)
ACTH, CORT	(A) and (B) blood (C) pituitary-derived cells	(A) *ip injection*: acute 0.3–3 g/kg	(A) 15-min postinjection	Anti-CRF serum, CRF (0.004–2.5 nM)	Rats: Sprague-Dawley (male) – adult	(A) Lowest dose of ethanol (0.3 g/kg) increased CORT but not ACTH levels, while 1 and 3 g/kg induced high levels of both hormones. Previous administration of anti-CRF serum abolished ethanol-induced ACTH release. (B) Higher concentrations of ethanol increased CORT secretion upon immediate withdrawal. (C) Acute exposure to 0.2% ethanol failed to affect CRF-induced ACTH release, but prolonged exposure (24 h) declined CRF-induced ACTH response	Rivier et al. (41)
		(B) *Vapor chamber*: 7 days (various concentrations)	(B) Immediately after removal from chamber				
		(C) *Cell culture incubation*: exposure to acute ethanol (during incubation with CRF – 4 h) or prolonged ethanol (24 h prior to CRF incubation)	(C) Immediately after incubation				
ACTH, CORT	Blood	*ip injection*: acute 1–3 g/kg	30 min–4 h		Rats: Sprague-Dawley – adult	*Acute ip and ig injection* of ethanol (2 and 3 g/kg) increased plasma ACTH levels, with peak release 30 min after injection; returning to basal levels in 180 min. *Liquid diet*: ethanol-fed rats presented lower levels of ACTH than pair-fed animals, but both groups presented similar plasma CORT response	Ogilvie et al. (167, 168)
		*Intragastric injection*: acute 1–3 g/kg					
		*Liquid diet*: 4 or 6 nights (12 h/day)					
ACTH, CORT	Blood	*Oral gavage* (1 ml, 75% ethanol)^a^	0–1 h		Rats: Wistar (male) – adult	Ethanol increased plasma ACTH (peak at 16 min) and CORT levels (peak at 5 min and elevation remains for 60 min)	Laszlo et al. (169)
ACTH, CORT	Blood	*Vapor chamber*: 7 days	3 h	iv CRF (0.3–10 μg) or footshock (1.0 mA, 0.5 s, 2 shocks/min; during 10 min)	Rats: Sprague-Dawley (male) – adult	At 3-h withdrawal, ethanol-exposed rats showed higher levels of ACTH and CORT. After CRF stimulation, controls show dose-dependent increases in ACTH levels, while ethanol-exposed rats showed increases in ACTH with no dose dependency. At the highest CRF dose, ethanol-exposed rats showed lower ACTH response than controls. Footshock induced similar ACTH response in both groups	Rivier et al. (170)
ACTH, CORT	Blood, pituitary	*Vapor chamber*: 7 days during the second gestational week. Pups were fed by foster mothers and euthanized at 21 days old. Pituitary maintained in short-term culture	28 days	Exposure to inescapable shocks (1.5 mA, 1 s, 25 times over 10 min) when pups were 21 days old, prior to euthanasia; CRF (0.01–100 nM)	Rats: Sprague-Dawley – prenatal/pup	Ethanol exposure during the second week of gestation potentiated plasmatic ACTH but not CORT response to shock stress. The ACTH released from the pituitary of ethanol-exposed pups was decreased after 3 h incubation with CRF and increased after incubation with higher CRF dose, when compared with control pups	Lee et al. (171)
ACTH, CORT, POMC (mRNA)	Blood, pituitary, and hypothalamus	*Oral gavage*: (4.5 g/kg/day) – 1 or 14 days	30 min after last adm		Rats: Fischer (male) – adult	Ethanol-induced acute increases in ACTH and CORT levels. After 14 days, ethanol’s effects on ACTH were abolished and CORT responses were reduced. Neither protocol changed POMC mRNA levels in pituitary. Hypothalamic POMC mRNA levels were reduced after acute, but not chronic ethanol	Zhou et al. (172)
ACTH, CORT; POMC (mRNA)	Blood, pituitary	*Liquid diet* (gestational): ethanol (~13 g/kg/day) during all pregnancy (withdrawal upon birth)	~4 months (exposure during prenatal period; tissue collected as adults)		Rats: Sprague-Dawley (male and female) – prenatal/adult	Prenatal ethanol exposure failed to affect plasmatic CORT and ACTH levels, or pituitary POMC mRNA	Glavas et al. (173)
ACTH, POMC (hnRNA and mRNA)	Blood, pituitary	*ip injection*: acute (3 g/kg)	0–60 min after ethanol adm	CRF antibody (0.4 ml/kg)	Rats: Sprague-Dawley (male) – adult	*ACTH*: both ip and icv ethanol injection induced increased plasma ACTH levels, a response that was prevented by anti-CRF antibody pretreatment. *POMC*: pituitary POMC primary transcript is increased 15, 30, and 60 min after ethanol ip and 15 min after ethanol icv. Anti-CRF antibody attenuated ethanol-induced POMC transcription	Lee et al. (174)
		*icv injection*: (5 μl)					
CORT	Blood	*Vapor chamber*: 6 h daily, during 2 or 8 days (in adolescence)	Immediately after removal from chamber, ranging from 1.5 to 5.5 h of vapor exposure		Rats: Sprague-Dawley (male) – adolescent	On the 2nd day of ethanol vapor exposure, adolescent rats presented increased levels of CORT after 3.5 and 5.5 h of ethanol vapor. On the 8th day, CORT levels were increased after exposure of 4.5 and 5.5 h to ethanol vapor	Logrip et al. (175)
CRF (hnRNA and mRNA)	Parvicellular PVN	*Ip injection*: acute (3 g/kg)	3 h	Astressin (non-selective CRF receptor antagonist; 15 μg, icv)	Rats: Sprague-Dawley (male) – adult	CRF heteronuclear RNA was increased after ethanol, as well as the combination of ethanol + astressin. CRF mRNA levels were unaffected	Lee and Rivier (176)
CRF (hnRNA and mRNA)	Parvicellular PVN	*Ip injection*: acute (3 g/kg)	20 min–3 h		Rats: Sprague-Dawley (male) – adult	CRF heteronuclear RNA was increased at 20 and 40 min after ethanol administration. CRF mRNA levels were unaffected	Rivier and Lee (164)
CRF (hnRNA and peptide)	PVN, median eminence (ME)	*Intragastric injection*: daily (4.5 g/kg), 3 days of treatment, ethanol challenge 7 days later	0–1 h		Rats: Sprague-Dawley (male) – adult	In PVN: ethanol pretreatment did not affect basal CRF hnRNA levels. Preexposure to ethanol reduced ethanol-induced upregulation of CRF hnRNA. In medial eminence (external zone): reduced CRF peptide levels in rats with prior ethanol history (after 7 days withdrawal)	Lee et al. (177, 178)
CRF (hnRNA)	Parvicellular PVN	*Icv injection*: acute (5 μl)	0–60 min after ethanol adm		Rats: Sprague-Dawley (male) – adult	PVN CRF hnRNA is increased 30 min after ethanol treatment	Lee et al. (174)
CRF (mRNA)	BLA	*Intermittent ethanol drinking*: daily access to one-bottle ethanol for 1 h, during 18 days, under water restriction condition. Intake ~2.5 g/kg/day for adolescents; ~2.3 g/kg/day for adults	60 days		Rats: Long-Evans (male) – adolescent – adult	BLA pre-pro-CRF mRNA levels were decreased in rats exposed to ethanol during adulthood, but not in those exposed during adolescence	Falco et al. (179)
CRF (mRNA)	CeA	*Liquid diet*: for 10–12 days (intake ~11.5 g/kg/day)	Immediately after ethanol removal		Rats: Sprague-Dawley (male) – 120 g (~5 weeks old)	Ethanol liquid diet increased pre-pro-CRF mRNA levels in CeA	Lack et al. (23)
CRF (mRNA)	CeA	*Vapor chamber*: intermittent exposure (8 h ethanol vapor/8 h air), for 8 on/off cycles. Ethanol (1.6 g/kg, ip) injected prior to every ethanol vapor session. Stress-induced ethanol self-adm (60-min session for 4 days; 120 min in the last session)	Immediately after removal from ethanol exposure; 2 weeks withdrawal; 2 weeks withdrawal + 4 h after the final self-adm session	4-h food restriction prior to ethanol self-adm sessions	Mice: C57BL/6N (male) – adult	Immediately after ethanol vapor exposure, there were no changes in CeA CRF mRNA levels. After 2 weeks of withdrawal, CeA CRF mRNA levels were increased, with further increases 4 h after the last drinking session	Eisenhardt et al. (180)
CRF (mRNA)	CeA, BNST	*Vapor chamber*: 7 weeks of ethanol exposure (17 h/day)	3 weeks		Rats: Wistar (male) –adult	Chronic ethanol exposure increased CRF mRNA levels in CeA, but not in BNST	Sommer et al. (18)
CRF (mRNA)	Hypothalamus	*Oral gavage* (4.5 g/kg/day) – 1 or 14 days	30 min after final adm		Rats: Fischer (male) – adult	No changes in CRF mRNA levels after acute or chronic ethanol	Zhou et al. (172)
CRF (mRNA)	Hypothalamus	*Vapor chamber*: 7 days during the second gestational week. Pups were fed by foster mothers and euthanized at 21 days old	28 days		Rats: Sprague-Dawley – prenatal/pup	Ethanol exposure during the second week of gestation increased hypothalamic CRF mRNA levels in the offspring	Lee et al. (171)
CRF (mRNA)	Parvicellular PVN	*Liquid diet* (gestational): ethanol (~13 g/kg/day) during all pregnancy (withdrawal upon birth)	~4 months (exposure during prenatal period; tissue collected as adults)		Rats: Sprague-Dawley (male and female) – prenatal/adult	Prenatal ethanol exposure failed to affect CRF mRNA levels in parvicellular PVN in males and females, when adults	Glavas et al. (173)
CRF (mRNA)	Parvicellular PVN	*Vapor chamber*: 7 days	Immediately after removal from chamber		Rats: Sprague-Dawley (male) – adult	Increased CRF mRNA levels in PVN after 3 or 7 days of ethanol vapor exposure	Rivier et al. (170)
CRF (mRNA)	PVN	*Vapor chamber*: 6 h daily, during 15 days (in adolescence). Also, 20 days later, rats received intragastric ethanol (4.5 g/kg) challenge	2 h after ethanol challenge (adulthood)		Rats: Sprague-Dawley (male) – adolescent/adult	Rats exposed to ethanol vapor during adolescence presented decreased levels of CRF mRNA in PVN after ethanol challenge in adulthood	Allen et al. (181)
CRF (mRNA)	PVN	*Vapor chamber*: 6 h daily, during 8 or 15 days (in adolescence). Also, 20 days later, rats received intragastric ethanol (4.5 g/kg) challenge	6 h after 8 or 15 days of vapor exposure (adolescence); or 2 h after ethanol challenge (adulthood)		Rats: Sprague-Dawley (male and female) – adolescent/adolescent-adult	During adolescence, there were no differences in PVN CRF mRNA levels after ethanol vapor exposure. Adolescent ethanol vapor exposure also failed to affect CRF mRNA levels in adult rats exposed to an ethanol challenge after 20 days of withdrawal	Logrip et al. (175)
CRF (peptide)	Amygdala	*Liquid diet*: 2–3 weeks	2–12 h		Rats: Wistar (male) –adult	10–12 h of withdrawal increases CRF immunoreactivity in amygdala dialyzates	Merlo-Pich et al. (182)
CRF (peptide)	BNST	*Liquid diet*: ethanol for 2 weeks, ~10 g/kg/day. Diet removal for 7.5 h + reexposure to ethanol diet	0–7.5 h		Rats: Long-Evans (male) – adult	Chronic ethanol had no effect on BNST CRF baseline levels (while still exposed to alcohol). During withdrawal, only ethanol-fed rats showed elevated levels of extracellular CRF in BNST, from 4.5 to 7.5 h. Upon reexposure to ethanol, CRF levels returned to baseline. Ethanol-fed animals exposed to control diet after abstinence, presented even further increases in CRF levels	Olive et al. (183)
CRF (peptide)	CeA	*Ip injection*: acute (2–2.8 g/kg)	Immediately – 180 min		Rats: Sprague-Dawley (male) – adult	Doses of 2.4 and 2.8 g/kg induced increased release of CRF in the CeA 120 min after administration, the effect was sustained until 180-min postinjection	Lam and Gianoulakis (184)
CRF (peptide)	CeA, VTA	*Drinking in the dark*: 1 cycle = 2-h ethanol access for 3 days, 4-h access in the 4th day. 3 days of withdrawal between cycles	0–24 h		Mice: C57BL/6J (male) – adult	CRF immunoreactivity was increased in CeA immediately after 1 or 6 cycles of drinking; in VTA only after 1 cycle. CRF immunoreactivity was also increased 18–24 h after 3 cycles of drinking	Lowery-Gionta et al. (73)
CRF (peptide)	Hypothalamus	*Drinking in the dark* (prenatal exposure): two-bottle choice (ethanol in saccharin or saccharin alone), 4 h/day during all gestational period. Upon birth, dams were gradually weaned out of ethanol over 6 days	40–50 days (prenatal exposure; euthania in late adolescence)		Mice: C57BL/6J (male) – prenatal/adolescent	Increased levels of CRF immunoreactivity in the hypothalamus of mice with prenatal ethanol exposure	Caldwell et al. (185)
CRF (peptide)	Hypothalamus	*Liquid diet*: 2 weeks. Acute ethanol exposure in hypothalamic superfusion (various concentrations)	Immediately after ethanol removal		Rats: Sprague-Dawley (male) – adult	Chronic ethanol exposure increased CRF release pulse frequency in a hypothalamic *in vitro* preparation. CRF content in hypothalamus was lower in ethanol-exposed rats than in controls. Incubation with acute ethanol produced dose-dependent increases on CRF release from hypothalamus, effects that were blunted in ethanol diet-exposed rats. Potassium-stimulated CRF release was also reduced in ethanol preexposed rats	Redei et al. (166)
CRF (peptide)	Hypothalamus	*Oral gavage* (1 ml, 75% ethanol)^a^	0–1 h	Cycloheximide (protein synthesis inhibitor; 10 or 30 mg/kg, ip)	Rats: Wistar (male) – adult	Ethanol increased hypothalamic CRF contents after 30 and 60 min. Cycloheximide reduced ethanol-induced CRF levels	Laszlo et al. (169)
CRF (peptide)	Median eminence, hypothalamus	*Vapor chamber*: 7 days (various concentrations of ethanol)	Immediately after removal from vapor chamber		Rats: Sprague-Dawley (male) – adult	Higher doses of ethanol reduced CRF immunoreactivity in median eminence, but not in hypothalamus	Rivier et al. (41)
CRF (promoter, mRNA and peptide)	Hypothalamic cell culture	*Ethanol incubation* (25 mM, during 1–4 h)	Immediately after ethanol incubation	Forskolin (adenylyl cyclase activator; 25 μM); PKA inhibitors H89 (10 μM), Rp-cAMP (250 μM)	Hypothalamic cells derived from Sprague-Dawley rats (6–7 days old)	Acute ethanol incubation increased CRF peptide levels (after 1 and 4 h), increased CRF mRNA (peak at 1 h incubation), and increased CRF promoter activity (after 2 h). These effects were potentiated with the combined incubation of ethanol and forskolin, except for ethanol-induced CRF mRNA, which was slightly blunted by forskolin. PKA inhibitors abolished the effects of ethanol incubation	Li et al. (186)
CRF-BP (mRNA)	CeA	*Liquid diet*: for 10–12 days (intake ~11.5 g/kg/day)	Immediately after ethanol removal		Rats: Sprague-Dawley (male) – 120 g (~5 weeks old)	No alterations in CRF-BP in the CeA after chronic ethanol exposure	Lack et al. (23)
CRFR1 (mRNA)	Amygdala	*Extended ethanol drinking*: three-bottle choice (water, ethanol 5%, and ethanol 10%) during 70 days, followed by 2 weeks of withdrawal and reexposure to three-bottle choice for 2 weeks. Then, quinine-adulterated ethanol for 2 weeks, followed by ethanol reexposure for 1 week	Immediately after the final ethanol exposure		Mice: Swiss (male) – adult	Mice with moderate ethanol intake profile (± 9.4 g/kg/day) presented increased CRFR1 mRNA levels in amygdala, while animals with high (± 11.5 g/kg/day) and low (± 5.2 g/kg/day) ethanol intake profile showed no alterations in CRFR1 mRNA	Correia et al. (83)
CRFR1 (mRNA)	Amygdala	*Operant self-administration*: after training lever-pressing for saccharin, 3 days of access to ethanol + saccharin solution (30-min session/day; ~1 g/kg ethanol)	20 days		Rats: Wistar (male) – adult	No correlations between alcohol consumption and CRFR1 mRNA expression in the amygdala	Pickering et al. (187)
CRFR1 (mRNA)	CeA, MeA, BLA	*Vapor chamber*: intermittent exposure (8 h ethanol vapor/8 h air), for 8 on/off cycles. Ethanol (1.6 g/kg, ip) injected prior to every ethanol vapor session. Stress-induced ethanol self-adm (60-min session for 4 days; 120 min in the last session)	Immediately after removal from ethanol exposure; 2 weeks withdrawal; 2 weeks withdrawal + 4 h after the final self-adm session	4-h food restriction prior to ethanol self-adm sessions	Mice: C57BL/6N (male) – adult	Immediately after ethanol vapor exposure, CRFR1 mRNA levels were decreased in CeA but increased after 2 weeks of withdrawal and 4 h after the last self-administration session. In MeA and BLA, CRFR1 levels were also increased after withdrawal and after the drinking session	Eisenhardt et al. (180)
CRFR1 (mRNA)	CeA, MeA, BLA, BNST	*Vapor chamber*: 7 weeks of ethanol exposure (17 h/day)	3 weeks		Rats: Wistar (male) – adult	Chronic ethanol exposure increased CRFR1 mRNA in BLA and MeA, but not CeA and BNST	Sommer et al. (18)
CRFR1 (mRNA)	CeA, MeA, BLA, Nacc, Cg	*Two-bottle choice*: continuous access to ethanol and water, 24 h/day for 15 days	Immediately after ethanol removal		Rats: alcohol-preferring (msP) (male) – adult	Chronic ethanol consumption decreased CRFR1 mRNA levels in CeA, MeA, and NAcc, but not in BLA or Cg	Hansson et al. (188)
CRFR1 (mRNA)	DRN	*Drinking in the dark*: three daily 1-h sessions (2-h interval between sessions), 5 days/week, for 3 weeks (intake ~9 g/kg/day)	3 h		Rats: alcohol-preferring (P) (male) – adolescent	Increased CRFR1 mRNA in the DRN of ethanol binge-drinking rats	McClintick et al. (189)
CRFR1 (mRNA)	Hypothalamus	*Operant self-administration*: after training lever-pressing for saccharin, 3 days of access to ethanol + saccharin solution (30-min session/day; ~1 g/kg ethanol)	20 days		Rats: Wistar (male) – adult	Strong positive correlation of alcohol consumption and hypothalamic CRFR1 mRNA expression	Pickering et al. (190)
CRFR1 (mRNA)	Parvicellular PVN	*Intragastric injection*: daily (4.5 g/kg), 3 days of treatment, ethanol challenge 7 days later	3 h		Rats: Sprague-Dawley (male) – adult/adult	Ethanol pretreatment did not affect basal CRFR1 expression. Preexposure to ethanol reduced ethanol-induced upregulation of CRFR1 mRNA	Lee et al. (177, 178)
CRFR1 (mRNA)	Pituitary	*Liquid diet* (gestational): ethanol (~13 g/kg/day) during all pregnancy (withdrawal upon birth)	~4 months (exposure during prenatal period; tissue collected as adults)		Rats: Sprague-Dawley (male and female) – prenatal/adult	Prenatal ethanol exposure reduced CRFR1 mRNA levels in the anterior pituitary of male but not female rats	Glavas et al. (173)
CRFR1 (mRNA)	Pituitary	*Oral gavage* (4.5 g/kg/day) – 1 or 14 days	30 min after final adm		Rats: Fischer (male) – adult	Acute, but not chronic, ethanol reduced CRFR1 mRNA in the anterior pituitary	Zhou et al. (172)
CRFR1 (mRNA)	PVN, amygdala	*Ip injection*: acute (3 g/kg)	3 h	Astressin (non-selective CRF receptor antagonist; 15 μg, icv)	Rats: Sprague-Dawley (male) – adult	Ethanol increased CRFR1 mRNA in parvicellular PVN, an effect not prevented by astressin. In magnocellular PVN, CRFR1 mRNA is increased after combined ethanol plus astressin administration. No ethanol-induced changes in amygdala CRFR1 mRNA levels	Lee and Rivier (176)
CRFR2 (mRNA)	Amygdala	*Operant self-administration*: after training lever-pressing for saccharin, 3 days of access to ethanol + saccharin solution (30-min session/day; ~1 g/kg ethanol)	20 days		Rats: Wistar (male) – adult	No correlations between alcohol consumption and CRFR2 mRNA expression in the amygdala	Pickering et al. (187)
CRFR2 (mRNA)	CeA, MeA, BLA	*Vapor chamber*: intermittent exposure (8 h ethanol vapor/8 h air), for 8 on/off cycles. Ethanol (1.6 g/kg, ip) injected prior to every ethanol vapor session. Stress-induced ethanol self-adm (60-min session for 4 days; 120 min in the last session)	Immediately after removal from ethanol exposure; 2 weeks withdrawal; 2 weeks withdrawal + 4 h after the final self-adm session	4-h food restriction prior to ethanol self-adm sessions	Mice: C57BL/6N (male) – adult	Immediately after ethanol vapor exposure, there were no effects on CRFR2 mRNA levels in CeA. After 2-week withdrawal, no changes were observed in CRFR2 mRNA levels in CeA, MeA, and BLA. Ethanol self-administration increased CRFR2 mRNA in CeA and BLA, but not in MeA in ethanol-exposed mice, relative to air-exposed controls	Eisenhardt et al. (180)
CRFR2 (mRNA)	CeA, MeA, BLA, BNST	*Vapor chamber*: 7 weeks of ethanol exposure (17 h/day)	3 weeks		Rats: Wistar (male) – adult	Chronic ethanol exposure decreased CRFR2 mRNA levels only in BLA	Sommer et al. (18)
CRFR2 (mRNA)	DRN	*Drinking in the dark*: three daily 1-h sessions (2 h interval between sessions), 5 days/week, for 3 weeks (intake ~9 g/kg/day)	3 h		Rats: alcohol-preferring (P) (male) – adolescent	Decreased CRFR2 mRNA in the DRN of ethanol binge-drinking rats	McClintick et al. (189)
CRFR2 (mRNA)	Hypothalamus	*Operant self-administration*: after training lever-pressing for saccharin, 3 days of access to ethanol + saccharin solution (30-min session/day; ~1 g/kg ethanol)	20 days		Rats: Wistar (male) – adult	Strong positive correlation of alcohol consumption and hypothalamic CRFR2 mRNA expression	Pickering et al. (190)
CRFR2 (mRNA)	VMH, SON	*Ip injection*: acute (3 g/kg)	3 h		Rats: Sprague-Dawley (male) – adult	CRFR2 mRNA in VHM or SON is not affected by ethanol	Lee and Rivier (176)
CRFR2 (protein)	LS, DRN	*Ip injection*: chronic (2 g/kg, daily), 0, 1, or 7 days	24 h		Mice: C57BL/6J (male) – adult	Higher CRFR2 binding in dorsomedial DR after 7 days of ethanol exposure. No changes in LS	Weitemier and Ryabinin (191)
POMC (mRNA)	Hypothalamus	*Operant self-administration*: after training lever-pressing for saccharin, 3 days of access to ethanol + saccharin solution (30-min session/day; ~1 g/kg ethanol)	20 days		Rats: Wistar (male) – adult	No correlations between alcohol consumption and hypothalamic POMC mRNA expression	Pickering et al. (190)
Ucn1	npEW, LS, DRN	*Ip injection*: chronic (2 g/kg, daily), 0, 8, or 15 days	20 min		Mice: C57BL/6J and DBA/2J (male) – adult	Repeated ethanol exposure (8 or 15 days) had no effect on Ucn1 cell count in npEW but reduced Ucn1 fibers in the LS and DR (DR only at 8 days). No strain differences	Weitemier and Ryabinin (191)

*^a^Estimated dose in the range of 3.0–3.5 g/kg ethanol*.

*ip, intraperitoneal; icv, intracerebroventricular; iv, intravenous; CeA, central nucleus of the amygdala; MeA, medial nucleus of the amygdala; BLA, basolateral amygdala; DRN, dorsal raphe nucleus; PVN, paraventricular nucleus of the hypothalamus; Cg, cingulate gyrus; BNST, bed nucleus of the stria terminalis; LS, lateral septum; npEW, Edinger–Westphal nucleus; VMH, ventromedial hypothalamus; SON, supraoptic nucleus; VTA, ventral tegmental area; NAcc, nucleus accumbens*.

As shown in Table 3, acute alcohol exposure, whether administered ip or by gavage, promotes activation of the HPA axis, as evidenced by dose-dependent increases on plasma ACTH and corticosterone with doses of 1 g/kg and higher [e.g., Ref. (41, 164, 169, 172)]. Increases in ACTH can also be elicited with icv administration of alcohol (174) and can be shown *in vitro* in a hypothalamus–pituitary preparation (166). Importantly, alcohol’s actions on ACTH and corticosterone release seem to be primarily mediated by alcohol-induced CRF activation in the hypothalamus [see review by Rivier (192)]. Pretreatment with antagonists of CRF receptors as well as with anti-CRF antibody, both prevent or attenuate alcohol-induced increases in ACTH and corticosterone [e.g., Ref. (164, 165, 174)]. Consistent with this hypothesis, heteronuclear RNA (precursor of mRNA) for CRF and CRF peptide levels are reliably increased in the hypothalamus after acute alcohol administration (164, 169, 174, 176). Hypothalamic CRF mRNA levels are not as reliably affected by acute alcohol, perhaps due to the kinetics of transcription processes (172, 176). In hypothalamic cell culture, alcohol incubation produced increases in CRF mRNA, CRF promoter activity, as well as CRF peptide, in a process that requires adenylate cyclase-PKA signaling (186). Extra-hypothalamic sites, such as the central amygdala, also show augmented release of CRF peptide as a result of acute alcohol administration (184). Concerning CRF receptors, mRNA levels may be affected by acute alcohol exposure, but results are scarce and inconsistent (172, 176), as shown in Table 3.

Chronic or repeated alcohol exposure promote less consistent changes on CRF/urocortin function, when components of the CRF system are analyzed under alcohol influence or *immediately* (within 60 min) after removal from alcohol exposure. Some reports suggest the development of tolerance in HPA axis responses after repeated alcohol treatment, with lower ACTH and corticosterone responses to alcohol [e.g., Ref. (172, 175); but see Ref. (41, 167, 168)]. Concerning CRF contents in the hypothalamus and/or pituitary, there are reports of no changes (41, 172), decreases (41, 166, 177, 178), or increases (170) in CRF peptide, mRNA, and/or hnRNA after chronic alcohol. For example, in the PVN, alcohol-induced upregulation of CRF hnRNA and alcohol-induced CRF release were reduced in rats with a history of alcohol exposure (166, 177, 178). In the CeA, repeated alcohol was reported to increase detection of CRF mRNA or immunoreactivity [(73, 193); but see Ref. (188)], although a lack of alteration was also found (180). In amygdalar nuclei, a reduction in CRFR1 gene expression, but not CRFR2, was detected after alcohol vapor exposure (180) or after 15 days of voluntary of alcohol drinking in an alcohol-preferring strain (188). After extended access to alcohol for over 90 days, only mice with a profile of moderate alcohol intake showed increased CRFR1 mRNA in the amygdala, which was not the case for mice with high or low intake profiles (83). In the BNST, no changes in CRF immunoreactivity were seen while animals still had access to alcohol in a liquid diet, but increases in CRF emerged after 4 h into withdrawal from alcohol (183). Interestingly, repeated alcohol failed to change the number of urocortin1 cells in the Edinger–Westphal nucleus, but it decreased urocortin1 fibers to the lateral septum and the DRN (191).

Short-term withdrawal (here defined as an arbitrary window of 1–24 h after the final alcohol exposure) from repeated/chronic alcohol administration is usually associated with increased anxiety-like behavior and escalated alcohol self-administration, as discussed in previous sections. Acute withdrawal (i.e., 3 h) from a 7-day alcohol vapor exposure was characterized by increased plasma ACTH and corticosterone levels, and also associated with increased CRF mRNA levels in the PVN (170). However, maximal CRF-mediated ACTH response was reduced in alcohol-exposed rats (170). A different study showed that after 1-day withdrawal from chronic alcohol liquid diet, CRF immunoreactivity was not changed in the hypothalamus but was reduced in the amygdala, hippocampus, and frontal cortex (194). These changes were also accompanied by lower plasma corticosterone concentrations at acute withdrawal. In a classic study on acute alcohol withdrawal, dialyzate CRF levels in the amygdala are shown to increase in great proportions throughout the first 12 h of withdrawal from chronic alcohol vapor exposure (182). Increases in CRF immunoreactivity in CeA are also observed after repeated cycles of binge alcohol drinking in mice, persisting for 18–24 h into alcohol withdrawal (73). In another stress-related region, the BNST, CRF peptide levels increase after 4.5 h into withdrawal from an alcohol liquid diet. Remarkably, CRF levels in the BNST return back to baseline levels when rats regain access to the alcohol-containing liquid diet (183). However, these effects of acute alcohol withdrawal may be age-dependent. Exposure to alcohol vapor or binge drinking for 8–15 days in adolescent rats produces no changes in CRF transcripts or peptides in the PVN (175, 195), although a reduced CRF immunoreactivity in the CeA is observed (195). As shown in Table 3, a few studies also reported changes in CRF receptors’ transcripts after short-term alcohol withdrawal in the PVN (177, 178) and the DRN (189), as well as in CRFR2 binding levels in the DRN (191).

Finally, critical adaptations in the CRF/urocortin are recruited during protracted abstinence from chronic alcohol exposure (i.e., at least 7 days of withdrawal, but usually longer than 2 weeks). For example, rat’s exposure to repeated alcohol administrations followed by a 7-day withdrawal, show reduced CRF peptide levels in the median eminence of the hypothalamus, despite no changes in CRF or CRFR1 transcripts in the PVN (177, 178). However, animals preexposed to the drug show a blunted upregulation of CRF and CRFR1 transcripts in the PVN in response to an alcohol challenge (177, 178). These data, and others, support a dampened function of HPA axis function after chronic alcohol followed by prolonged withdrawal, which is also supported by evidence of low baseline corticosterone levels after 3-weeks withdrawal from chronic alcohol [e.g., Ref. (194)]. As shown in Table 3, prenatal exposure to alcohol also dysregulates elements of the HPA axis in adolescent and adults animals, although differences in neuroadaptations arise according to the duration and methodology of *in utero* alcohol exposure [e.g., Ref. (171, 173, 185)]. Chronic exposure to alcohol during adolescence may lead to reduced HPA axis response to alcohol in early adulthood, as shown by reduced CRF mRNA in the PVN (181). However, such outcome was not observed when adolescents were exposed to alcohol as binge drinking (195). Thus, recruitment of neuroadaptive changes in CRF/urocortin elements in the HPA axis depend on age of alcohol exposure, the duration and protocol for alcohol exposure (vapor, liquid diet, oral intake), as well as age at time of assessment of CRF/urocortin targets.

In the amygdala, and particularly within the CeA, there is consistent evidence of upregulated CRF signaling after protracted withdrawal from chronic alcohol exposure. For example, there are increased levels of CRF transcript in the CeA (18, 180) or immunoreactivity for CRF in the amygdala (194) after 3 or more weeks of withdrawal from alcohol. These changes are accompanied by increased CRFR1 gene expression within the CeA and/or in other amygdalar nuclei (BLA, MeA). Changes in CRFR2 transcripts in the amygdala were less consistent within different studies (18, 180). One study also reported a decrease in CRF mRNA in the BLA in adult, but not adolescent rats after 18 days of restricted access to alcohol drinking followed by a 60-day withdrawal (179). Although BNST levels of CRF seem to be upregulated during acute withdrawal (183), BNST may not be particularly engaged in protracted abstinence, as no changes in transcripts for CRF or CRFR1 were seen in this region (18).

In summary, alcohol exposure importantly impacts CRF/urocortin components and function, both within the HPA axis and in extra-hypothalamic sites. In general, acute alcohol reliably upregulates CRF transcripts and/or peptide within the hypothalamus, promoting activation of the HPA axis with increases in ACTH and corticosterone. When CRF/urocortin signaling is assessed shortly after chronic alcohol exposure, relevant but quite variable and inconsistent effects may be detected, depending on brain region, alcohol exposure protocol, and the age of animals. After protracted abstinence from alcohol, a more reliable profile emerges, characterized by hyperactivity of brain CRF signaling, particularly within the amygdala. Although the studies are limited, existing research suggests the involvement of urocortins as targets for alcohol’s effects, as well as changes in CRFR2 mRNA/protein levels, and therefore these molecules should be further investigated. Also, target brain regions were highly limited to the hypothalamus, amygdala, and BNST, with a few exceptions. A wider characterization of alcohol modulation of CRF/urocortin signaling should include the VTA, accumbens, DRN, prefrontal cortex, and other reward-related structures, which may contribute to our understanding of alcohol-induced neuroadaptations that influence alcohol reward and withdrawal.

## Involvement of CRF Systems in Human Alcoholism

This review will discuss a few human studies regarding HPA axis function in AUDs. For a vast discussion on this literature, we also recommend reviews by Lovallo (196); Rose et al. (163); Stephens and Wand (2). As described in animal studies, human studies confirm an activation of HPA axis by alcohol as demonstrated by increases in plasma cortisol after an acute alcohol administration (197). This cortisol response seems to require ACTH release from the hypophysis, as alcohol failed to increase cortisol levels in patients with lesions in the anterior hypophysis (197). In heavy drinkers and dependent subjects, there is an attenuation of alcohol-induced cortisol response (198), as well as reduced cortisol and ACTH levels upon CRF stimulation (199–201). Dependent individuals also show an attenuated cortisol response to moderate mental and physical stressors (202–205). Upon alcohol withdrawal, plasma cortisol is elevated during the first week, but with protracted abstinence, cortisol levels drop below normal range (206–208). Together, these studies suggest a blunted HPA function in response to drugs and stressors in alcoholics, supporting a contribution of the HPA axis in the transition to dependence (209).

Studies targeting the CRF system and alcohol in humans mostly rely on genetic approaches, with evidence of associations between AUDs and polymorphisms in genes encoding for CRFR1 and CRF-BP. Chen et al. (210) showed that variations in the CRFR1 gene are associated with alcohol dependence. In another study, patterns of alcohol consumption were assessed in a teenage sample, as well as in a sample of dependent adults. In teenagers with little to moderate alcohol exposure, polymorphisms in CRFR1 gene were associated with the amount of alcohol consumed, but not with the frequency of drinking episodes (211). In alcohol-dependent adults, an association was also detected between CRFR1 polymorphism and the amount of alcohol consumed (211). Different studies show gene–environment interactions, in which variants of the CRFR1 gene interact with a history of exposure to stressful events and predict heavy alcohol drinking (212, 213) and age of drinking initiation (213). On the other hand, protective effects for alcohol dependence were shown for a gene–environment interaction involving a CRFR1 haplotype and childhood sexual abuse in an Australian population (214).

Electroencephalogram phenotypes have been identified for anxiety-prone alcoholics (215, 216). A “linkage scan” indicated CRF-BP as a candidate gene for electroencephalogram profiles in two distinct populations. Further, genetic variations in CRF-BP were associated with anxiety disorders and AUDs (217). There is also evidence that polymorphisms in CRF-BP modulate stress-induced craving in heavy drinkers (218) and are involved in the relationship between negative reactivity to stress and negative consequences to alcohol consumption (219). Thus, CRF-BP gene variations may play particular a role in stress-related alcohol dependence. Furthermore, an interaction of polymorphisms in CRFR1 and CRF-BP genes predicts increased risk for AUDs in schizophrenic patients (220). Thus, genetic studies, so far, point to some polymorphisms in CRFR1 and CRF-BP genes, which can contribute to the susceptibility for AUDs.

## Summary and Perspectives

Increasing evidence suggests that a recruitment of CRF/urocortin mechanisms is more prominent in individuals with increased sensitivity to and/or vulnerability for alcohol-induced effects (5). After repeated and chronic exposure to alcohol, rats present hyperactive extra-hypothalamic CRF activity, as indicated by increases in CRF immunoreactivity and/or increases in mRNA for CRF and its receptors in amygdala nuclei and the BNST (18, 182, 183). Consistently, increased alcohol seeking and intake can be attenuated after the administration of antagonists to CRF type 1 receptors (systemically, icv or intra-amygdala), particularly in animals with previous alcohol history (5, 18, 81, 106, 109, 110) or in high alcohol-consuming mice (76). While blocking CRFR1 attenuates alcohol drinking, this effect can also be achieved with the activation of CRFR2 signaling, suggesting opposite roles for CRFR1 and CRFR2 in the modulation of excessive alcohol intake [e.g., Ref. (86, 105, 113)]. However, manipulation of CRF receptor signaling in different brain regions may reveal differential effects and interactions between CRFR1 and CRFR2 [e.g., Ref. (73, 99, 105, 115)]. Pharmacological studies start to unveil a role for both CRF receptors within brain reward pathways, as well as CRF-BP, as critical modulators of escalated alcohol drinking [e.g., Ref. (74, 84, 89, 115)]. Furthermore, the finding that different CRF mechanisms and pathways may be engaged during escalated drinking or alcohol withdrawal-related anxiety (99) gives rise to an intriguing path for further investigation. Moreover, CRF/urocortin signaling is also recruited during other alcohol-related effects, including alcohol-induced behavioral sensitization, alcohol-escalated aggression, and alcohol withdrawal-related anxiety. Determination of specific mechanisms for the engagement of CRF, urocortins, CRF-BP, and CRF receptors, after acute and chronic alcohol exposure still remain to be clarified. While effects of alcohol on the HPA axis, the amygdala, and other stress-related structures have been more widely characterized, promising contributions of CRF/urocortin signaling in brain reward pathways and other structures are starting to emerge. Finally, there is hope that new molecules targeting components of the CRF/urocortin system will be made available for testing in preclinical experimental settings and advancing to clinical trials, providing helpful therapeutic tools for the treatment of AUDs.

## Author Contributions

IQ and GM proposed and outlined the contents of the review. IQ, GM, LD, and CF wrote different sections of the text, prepared the corresponding tables, and organized the bibliography. IQ revised the manuscript text and tables. All authors carefully read, revised, and approved the entire text.

## Conflict of Interest Statement

The authors declare that the research was conducted in the absence of any commercial or financial relationships that could be construed as a potential conflict of interest.

## Footnotes

^1^This affirmation refers exclusively to locomotor sensitization induced by alcohol. Neurochemical sensitization of the dopamine reward system and sensitization to other behaviors and drugs (including cross-sensitization between alcohol and other drugs; withdrawal-induced sensitization of anxiety-like behaviors, etc.) have been extensively demonstrated in rats.
